# What is the most dangerous snake?

**DOI:** 10.1186/1678-9199-19-19

**Published:** 2013-08-27

**Authors:** Marshall D McCue

**Affiliations:** 1 St. Mary’s University, San Antonio, TX, USA

**Keywords:** Snakes, Snake bites, Snake venoms, Snake envenomation

## Abstract

Letter to Editor of Journal of Venomous Animals and Toxins.

## 

Snake venoms are complex mixtures of bioactive molecules that scientists can now physically separate and chemically characterize [1]. Interestingly, the snakes that possess these venoms remain among the most misrepresented animals outside of science. Primitive societies aware of the deleterious effects of venomous snake bites assigned mythological properties to these animals [2], but early naturalists are also responsible for spawning fallacious claims about snake venoms.

“Molecules of living germinal matter are thrown into the blood from the venom, and speedily grow into cells and multiply so that in a few hours millions are produced” [3].

“Melancholy is quickly changed into gay anticipations [upon ingestion of venom]… An ideot became improved in intellect” [4].

“It is reported that if the party live that is bitten, the snake will dye, and if the party dye that snake will live” [5].

Because of social and cultural inertia these and other misconceptions remain part of our popular culture – among these is the myth of the most dangerous snake. People who study venomous snakes are invariably asked the same question, “What is the most dangerous venomous snake?”. This question is impossible to answer without further clarification. Does the person want to know “Which snake species has the most venom?” or “Which is the deadliest snake species?”. The absolute volume of venom possessed by various species is fairly straightforward, albeit risky to quantify, yet is confounded by numerous factors including size, age, health, and method of extraction [6]. Nevertheless detailed tables of wet and dry extracted mass can be found across decades of literature [7].

Does the person want to know which species is the deadliest? Then it needs to be further clarified; deadliest for whom? Different animals demonstrate lethal sensitivities that vary by several orders of magnitude [8]. In general, ectothermic animals tend to be less sensitive to snake venoms [9] whereas some birds may have unusually high sensitivities to snake venoms; but there are countless exceptions to both of these trends. Moreover, some animals (e.g., wood rats, cotton rats) have clearly evolved physiological resistance to the venoms of sympatric snake species [10], but may have no particular resistance to the venoms of snakes with which they have not coevolved.

Laboratory animals like mice, rats, or guinea pigs, traditionally used in quantitative determinations of venom toxicity are inbred and may yield consistent measurement outcomes [11], but none of these are necessarily representative of wild animals or humans. Further confusion occurs when laboratory mammals are employed to score the toxicity of venoms from species that would never encounter mammalian predators or prey [12]. The mode of venom administration (i.e., intravenous, subcutaneous, intramuscular, or intraperitoneal) has a tremendous influence on the toxic action of venoms [13], and minimum lethal dosages may need to be several hundred times greater when delivered subcutaneously than those administered intravenously. Therefore, any extrapolations from contrived laboratory situations must be interpreted with caution.

Perhaps the person wants to know the snake species that is most deadly to humans. If so one would need to consider, “Which snake causes the most deaths?” or “Which snake is most likely to have a lethal bite?”. The answer to the former question is confounded by patterns of human population density and social habits. For example cobras are responsible for tens of thousands of deaths each year in south-central Asia, but a species that would otherwise be considered similarly dangerous may inhabit areas with low human densities (e.g., Mojave rattlesnake or inland taipan) and thus kill very few people [14]. Different species also have different propensities to offensively or defensively bite humans [15]. The answer to the latter question is confounded by the levels of emergency health care available to snakebite victims located in different geographic and/or demographic regions.

We may never objectively determine which snake is the most dangerous. My position is that the most dangerous snake is the one that just bit you.

## Competing interests

The author declares that there are no competing interests.

## References

[B1] Mackessy SP The field of reptile toxinology: snakes, lizards and their venoms In Handbook of venoms and toxins of reptiles 2009 CRC Press/Taylor & Francis Group: Mackessy SP. Boca Raton, FL 3 23

[B2] Klauber LM Rattlesnakes: their habits, life histories, and influence on mankind 1997 Los Angeles: University of California Press

[B3] Mitchell SW Experimental contributions to the toxicology of rattle-snake venom New York Medical Journal 1868 6 4 311

[B4] Wallace JW The therapeutic use of rattlesnake-venom The Medical Times Dec 1870 15 101 102

[B5] Mitchell SW The poison of the rattlesnake The Atlantic Monthly 1868 21 126 452 461

[B6] Macht DI Comparative toxicity of sixteen specimens of *Crotalus* venom Proc Soc Exp Biol Med 1937 36 1 499 501

[B7] Allen ER Maier E The extraction and processing of snake venom Copeia 1941 4 248 252

[B8] Noguchi H The action of snake venom upon cold-blooded animals 1904 Washington: Carnegie Institution of Washington

[B9] Andrade DV Abe AS Relationship of venom ontogeny and diet in *Bothrops* Herpetologica 1999 55 2 200 204

[B10] Perez JC Pichyangkul S Garcia VE The resistance of three species of warm-blooded animals to western diamondback rattlesnake (*Crotalus atrox*) venom Toxicon 1979 17 6 601 607 10.1016/0041-0101(79)90234-4 524385

[B11] Bolyn AE The standardization of snake venoms in mice in terms of hemorrhagic units J Bacteriol 1940 40 330 331

[B12] Sasa M Diet and snake venom evolution: can local selection alone explain intraspecific venom variation? Toxicon 1999 37 2 249 252 10078860 10.1016/s0041-0101(98)00121-4

[B13] Brown JH Toxicology and pharmacology of venoms from poisonous snakes 1973 Springfield: Charles C Thomas

[B14] McCue MD Enzyme activities and biological functions of snake venoms Appl Herpetol 2005 2 2 109 123 10.1163/1570754043492135

[B15] Hayes WK Herbert SS Rehling GC Gennaro JF Factors that influence venom expenditure in viperids and other snake species during predatory and defensive contexts In Biology of the vipers 2002 Eagle Mountain: Eagle Mountain Publ: Schuett GW, Hoggren M, Douglas ME, Greene HW

